# Factors predicting successful sperm retrieval in men with nonobstructive Azoospermia: A single center perspective

**DOI:** 10.1002/hsr2.727

**Published:** 2022-07-21

**Authors:** Abdulkareem Aljubran, Omar Safar, Adel Elatreisy, Raed Alwadai, Osama Shalkamy, Hassan Mohammed Assiri, Mamdoh Eskander, Adel Arezki, Ahmed Ibrahim

**Affiliations:** ^1^ Department of Urology Armed Forces Hospital Khamis Mushait Aseer Saudi Arabia; ^2^ Department of Urology, Faculty of Medicine Al‐Azhar University Cairo Egypt; ^3^ Department of Urology King Abdullah Hospital Bisha Aseer Saudi Arabia; ^4^ Department of Obstetrics and Gynecology, College of Medicine King Khalid University Abha Saudi Arabia; ^5^ Division of Urology, Department of Surgery McGill University Health Center Montreal Québec Canada

**Keywords:** Azoospermia, infertility, microsurgical testicular sperm extraction, nonobstructive, sperm retrieval, testicular sperm extraction

## Abstract

Predicting successful sperm retrieval is essential in counseling infertile men with Azoospermia.

**Objectives:**

To assess the predictors of successful sperm extraction in men with nonobstructive Azoospermia.

**Patients and Methods:**

A retrospective study included all patients with nonobstructive Azoospermia from January 2018 to May 2019. Subdivided into two groups, group I (negative sperm retrieval) and group II (positive sperm retrieval).

**Results:**

A total of 108 patients with a mean age of 36.8 ± 10 years were included. The rate of successful sperm retrieval was 47.2%. Group I included 57 patients (52.8%) with a mean age of 33.98 ± 6.18, and group II included 51 patients (47.2%) with a mean age of 40.04 ± 12.22 (*p* = 0.008). Follicular stimulating hormone (FSH) levels were significantly higher in group I (18.55 ± 13 vs. 7.97 ± 7.11; *p* < 0.004). Similarly, in group I, luteinizing hormone was significantly higher (11.4 ± 7.45 vs. 5.9 ± 4.4; *p* < 0.001). Age and FSH were the independent predictors of successful micro‐TESE. Additionally, successful pregnancies were 13.7% of patients, 28.6% of which gave rise to living birth.

**Conclusion:**

Patients' age and serum FSH are independent predictors of successful sperm retrieval for infertile men with nonobstructive Azoospermia; young patients with high FSH levels could have little chance of sperm retrieval.

## 1 INTRODUCTION

The prevalence of Azoospermia is nearly 10% among infertile men.^1^ Nonobstructive Azoospermia (NOA) caused by testicular failure and impaired sperm production represents approximately 60% of these cases.^2^

Patients with NOA are treated using microsurgical testicular sperm extraction (micro‐TESE) followed by intracytoplasmic sperm injection (ICSI).^3^ However, one of the significant challenges of this technique is our inability to predict the successful recovery of spermatozoa accurately. As a result, numerous ancillary tests such as testicular ultrasound, testicular biopsy, and hormonal markers have improved recovery rates.^4^ Though, none of the previous tests have proven to be effective. Furthermore, despite worrisome features, patients continue to undergo the extraction procedure.^5^

Almost half of the reported cases of NOA will have successful spermatozoa extraction with the help of micro‐TESE. Therefore, identifying patients with a high likelihood of achieving effective spermatozoa recovery is crucial in couples counseling.^6^, ^7^, ^8^ Nevertheless, researchers have yet to find a clinical or investigational finding that can accurately predict the outcome of micro‐TESE.^9^, ^10^ Previous studies have attempted to describe the relationship between testicular histopathology and the success rates of micro‐TESE, albeit with conflicting results.^11^, ^12^, ^13^, ^14^ At our center, the present study assessed the factors influencing successful sperm extraction in men with NOA.

## 2 PATIENTS AND METHODS

### 2.1 Study design

A retrospective study was performed on patients with NOA referred to the Uro‐Andrology clinic at Abha International hospital, Abha, KSA, between January 2018 and May 2019. The diagnosis of Azoospermia was based on at least two semen analyses. Patients were subdivided into two groups. Group, I included patients with negative sperm retrieval, and group II had those with positive sperm retrieval.

### 2.2 Data collection

A specialized checklist was prepared for the study, including the patient's age and focused clinical history, including a history of undescended testis, varicocele ligation, mumps orchitis, genitourinary infections, and exposure to gonadal toxin or chemo‐radiation. Laboratory data were collected regarding serum follicular stimulating hormone (FSH), serum luteinizing hormone (LH), serum testosterone, semen parameters, and Johnsen's score. Univariate and multivariate analyses were conducted to determine the predictors for successful sperm retrieval. The procedure was carried out as previously described.^13^

### 2.3 Surgical technique

Patients were done under spinal anesthesia and had surgical sperm retrieval using the micro‐TESE technique. A median raphe incision was made in the scrotum, the tunica vaginalis was opened, and the testis was delivered. An equatorial incision involving three‐quarters of the circumference of the testis was made using the surgical microscope. Micro‐dissection and exposure of the seminiferous tubules were performed, and testicular tissue was taken from the dilated tubules. Cryopreservation was used to preserve viable sperm for future use in ICSI. A histopathology specimen was sent for analysis and placed in Bouin's solution.

Testicular histology was classified into normal spermatogenesis, hypospermatogenesis (i.e., a reduction in the number of normal spermatogenetic cells), maturation arrest (i.e., absence of the later stages of spermatogenesis), and Sertoli cell‐only (SCO) (i.e., the lack of germ cells in the seminiferous tubules). For each testicular biopsy, Johnsen's score was determined.

### 2.4 Statistical analysis

We utilized the Statistical Package for Social Science (SPSS) software, version 27 (SPSS Inc.) for data analysis. Categorical variables were presented as frequency and percentage, whereas numeric variables were presented using mean, median, interquartile range (IQR), and standard deviation. A Chi‐square test was used to test the association between two categorical variables. At the same time, the Student *t* test was applied to assess for differences in the means of a continuous variable between two different groups. Since the distributions of age, FSH, LH, and testosterone were abnormally distributed (evidenced by the significant Shapiro–Wilk test, *p* < 0.001), nonparametric statistical tests were used for comparisons. Mann–Whitney test was used to compare the study groups. Statistical significance was determined at *p* < 0.05. Multivariate logistic regression analysis was used to define the predictors for sperm retrieval success in patients with NOA.

## 3 RESULTS

The study included 108 patients with NOA. Patient demographics and parameters are shown in Table 1. Group I included 57 patients (52.8%), and group II included 51 patients (47.2%). The average age of patients was 33.98 ± 6.18 and 40.04 ± 12.22 in groups I and II, respectively. The median age of patients in group I was significantly lower than that of patients in group II (median age of 33 [IQR: 20–37] and 35 [IQR: 32–43], respectively, *p* = 0.008).

**Table 1:** Summary statistics table for interval and ratio variables

Variable	*M*	*SD*	*n*	*SEM*	Min	Max	Skewness	Kurtosis
Serum LH	9.19	6.92	77	0.79	0.20	37.40	1.66	2.95
Serum FSH	14.21	12.12	78	1.37	0.60	49.50	1.18	0.72
Testosterone	11.03	32.54	72	3.83	1.20	280.00	8.00	63.68
Age	36.84	9.95	108	0.96	22.00	75.00	1.77	3.18
Undescended testis	0.05	0.21	108	0.02	0.00	1.00	4.32	16.65
Presence of varicocele	0.24	0.43	108	0.04	0.00	1.00	1.21	−0.53
volume	3.61	1.3	81	0.19	0.20	8.50	1.34	1.94
sperm count	119.08	1091.09	84	119.05	0.00	10000.00	0.85	1.58
Johnsen score	3.64	2.10	47	0.31	2.00	8.00	0.84	−0.59

*Note*: “‐” indicates the statistic is undefined due to constant data or insufficient sample size.

History of undescended testis was reported among five patients (4.6%) in group I. All the cases of undescended testis had negative sperm retrieval compared to a sperm retrieval rate of 50.5% in patients without undescended tests, *p* = 0.038. Additionally, 53.8% of the varicocele ligation cases and 45.1% of patients without a history of varicocele had positive sperm retrieval results. However, the difference was statistically insignificant, *p* = 0.437. No patients had a positive history of mumps‐orchitis, genitourinary infection, radiotherapy, chemotherapy, surgical procedures, or exposure to gonad toxins.

Serum FSH levels were significantly higher in group I than in group II (IQR: 7.8–25.9 vs. 3.1–10.1; median 17.1 vs. 6.2 *p* < 0.001). Similarly, serum LH levels were significantly higher in group I than in group II (IQR: 6.2–14.3 compared to 3.6–6.3 and median 9.4 vs. 4.4, respectively, *p* < 0.001). There was no significant difference between both groups in terms of seminal fluid volume (3.26 ± 1.43 vs. 3.7 ± 1.23; *p* = 0.17) and serum total Testosterone (3.3 ± 8.9 vs. 4.1 ± 12.3 *p* = 0.304) Table 2.

**Table 2:** Comparison of Azoospermia patients variables between group I (negative sperm extraction by TESE/micro TESE) and group II (positive sperm extraction by TESE/micro TESE)

variable	Group I	Group II	*p* value
Age (years)			
Median	33	35	0.008
IQR	20–37	32–43	
Mean rank	47.0	62.9	
Undescended tests			
No (*n* = 103)	52 (50.5)	51 (49.5)	0.038
Yes (*n* = 5)	5 (100)	0 (0.0)	
Varicocele			
No (*n* = 82)	45 (54.9)	37 (45.1)	0.437
Yes (*n* = 26)	12 (46.2)	14 (53.8)	
FSH	*N* = 46	*N* = 32	
Median	17.1	6.2	<0.001
IQR	7.8–25.9	3.1–10.1	
Mean rank	48.2	27.0	
LH	*N* = 46	*N* = 31	
Median	9.4	4.4	<0.001
IQR	6.2–14.3	3.6–6.3	
Mean rank	48.3	25.2	
Testosterone	*N* = 42	*N* = 30	
Median	5.6	6.8	0.304
IQR	3.3–8.9	4.1–12.3	
Mean rank	34.4	39.5	
Histopathology			
Hypospermatogenesis (*n* = 4)	3 (75%)	1 (25%)	0.559
Maturation arrest (*n* = 15)	13 (86.7%)	2 (13.3%)	
Sertoli cell only (*n* = 26)	24 (92.3%)	2 (7.7%)	
Semen parameters			
Volume	3.26 ± 1.43	3.7 ± 1.23	0.17
Ph	7.83 ± 1.18	7.87 ± 0.59	0.831

Abbreviations: FSH, follicular stimulating hormone; IQR, interquartile range; LH, luteinizing hormone; micro TESE, microsurgical testicular sperm extraction (micro‐TESE).

There was no statistically significant association between hypospermatogenesis, maturation arrest, and SCO from one side and the result of sperm extraction by surgical sperm retrieval techniques from the other side, *p* = 0.559. However, there was a clinical significance as 25% of patients with hypospermatogenesis had positive sperm extraction compared to 15.4% and 8.3% for maturation arrest and SCO, respectively. The most frequent Johnsen score in group one was a score of 2 (60%), followed by 5 (30%), 8 (7.7%), and 6 (2.5%).

Among the 51 cases of successful sperm extraction, pregnancy was achieved in 13.7% of cases, and 28.6% of these 13.7% were delivered a live baby.

Multivariate logistic regression analysis (Table 3) revealed that patient age and serum FSH were independent predictors for successful sperm retrieval in men with NOA. An increase in age by 1 year decreased the probability of a negative result by 9% (adjusted odds ratio [AOR] = 0.91; 95% confidence interval [CI] = 0.84–0.99, *p* = 0.023). Furthermore, an increase in serum FSH by one unit increased the probability of a negative result by 10% (AOR = 1.10; 95% CI = 1.01–1.20, *p* = 0.028).

**Table 3:** Predictors of sperm retrieval outcome: multivariate logistic regression analysis

	*B*	*SE*	AOR	95% CI	*p* value
Age (years)	−0.096	0.042	0.91	0.84–0.99	0.023
FSH	0.097	0.044	1.10	1.01–1.20	0.028
LH	0.115	0.075	1.12	0.97–1.30	0.123

*Note*: Variable of undescended testis was removed from the final model (not significant).

Abbreviations: AOR, adjusted odds ratio; B, slope; CI, confidence interval; FSH, follicular stimulating hormone; LH, luteinizing hormone; *SE*, standard error.

## 4 DISCUSSION

Testicular sperm extraction provides patients known for NOA the opportunity to have their biological children.^5^ However, only half of the reported cases of NOA will have successful spermatozoa extraction using micro‐TESE. Therefore, appropriate preoperative counseling regarding the likelihood of procedural success is essential to set realistic expectations for the infertile couple.^15^ The current study aims to evaluate the predictors of testicular sperm extraction outcomes in men with NOA.

In the present study, the rate of surgical retrieval of viable spermatozoa was 47.2%. A similar retrieval rate (50%) was previously reported in the literature.^6^, ^13^, ^16^ In comparison, our rate was considerably lower than the 65.5% successful retrieval rate reported by Eken and Gulec.^5^ Additionally, Schlegel observed an increase in spermatozoa retrieval rate from 45% to 63% using a micro‐dissection technique.^17^

Similar to previous studies, our study's FSH and LH levels were higher in micro‐TESE negative patients.^5^ However, there was no appreciable association between serum testosterone levels and the outcome of micro‐TESE. Eken and Gulec^5^ reported that, despite their overall correlation with the predominant spermatogenesis pattern, FSH levels might not predict specific areas of active spermatogenesis within the testis. Additionally, Ramasamy et al.^18^ concluded a nonstraightforward relationship between spermatogenesis and FSH levels in patients with NOA; in their study, the serum FSH level had a low predictive value for successful sperm retrieval. Furthermore, the plasma FSH concentrations are thought to be less accurate than testicular histopathology in predicting sperm retrieval.^13^ In a recently published meta‐analysis, FSH and testicular volume had low predictive values regarding successful micro‐TESE, while histopathological findings were considered a useful predictor.^12^ This notion is further supported by finding normal plasma FSH concentrations in many patients with maturation arrest.^19^ The differences observed between these studies may be attributed to variations in demographic characteristics.

Chen et al.^20^ Mentioned that An FSH cut‐off value of 13.7 mIU/ml discriminated between groups A and B with a sensitivity of 85.7% and a specificity of 87.0%. When the cut‐off value was increased to 19.4 mIU/ml, the sensitivity was 85.7%, and the positive predictive value could reach 100%. Interestingly, Elevated plasma levels of FSH (>19.4 mIU/ml) can preclude the presence of spermatogenesis with a probability of 100%.^20^ In our study, age, and serum FSH were significantly associated with surgical retrieval of spermatozoa. An increase in age by 1 year decreased the probability of a negative result by 9%. An increase in serum FSH by one unit increased the probability of a negative result by 10%; meanwhile, serum LH was not significantly associated with successful sperm retrieval. However, age and FSH cut‐off values were not done in this study.

Ramasamy et al.^18^ Mentioned in their nomogram to predict the probability of successful sperm retrieval in a micro‐TESE that the older age and higher FSH predict better chances of sperm retrieval with internal validation; the nomogram accuracy was 59.6%. The current study agrees with that nomogram in one point: older patients have a better chance for positive sperm retrieval; conversely, higher FSH has a low probability. We defined the young patients with high FSH levels who could have little chance of sperm retrieval.

Interestingly, Gnessi et al.^21^ concluded that semen and testicular histology were significant predictors of successful sperm retrieval. In contrast, age, semen, histology, and serum FSH levels were significant variables in predicting the time for sperm recovery.

Many studies have proven that the testicular biopsy is the strongest predictor of sperm retrieval.^13^, ^22^, ^23^, ^24^ Conversely, the current study revealed no association between testicular histopathological findings and the successful extraction of sperm via surgical retrieval techniques. Still, there was a clinical significance as 25% of patients with hypospermatogenesis had positive sperm extraction compared to 15.4% and 8.3% for maturation arrest and SCO, respectively. It can be explained by the different study populations included in the present study.

Similar to Sousa et al.^14^ and Abdel Raheem et al.,^13^ the presence of associated male pathologies was not predictive of retrieval success in the present study. However, all five cases of undescended testis had a failure of sperm retrieval. However, Negri L et al.^25^ azoospermia in patients with a history of cryptorchidism should not automatically be considered NOA: coexistence of spermatogenesis alteration and congenital seminal duct anomaly (the latter responsible for Azoospermia) is frequent. It could represent up to 60% of the cryptorchid azoospermic men.^25^ Also, a Relatively small sample size could explain this finding in our study.

In the current study, 25% of patients with hypospermatogenesis had positive sperm extraction compared to 15.4% and 8.3% for patients with maturation arrest and SCO, respectively. In comparison, Eken and Gulec^5^ successfully retrieved sperm in 96.5% of men with hypospermatogenesis and 42% of men with maturation arrest.^5^ Seo and Ko^26^ also observed a higher spermatozoa recovery rate among men with hypospermatogenesis and maturation arrest.

This study observed a 13.7% pregnancy rate among men with successful sperm extraction, 28.6% of whom delivered a live baby. Other studies reported higher pregnancy rates ranging between 26% and 44.6%.^5^, ^27^, ^28^, ^29^, ^30^, ^31^ Regarding the percentage of live births, Eken and Gulec^5^ reported 17.9%. Our reported rate is encouraging for men with NOA.

Hypoxia could interfere with spermatogenesis and enhance germ cell apoptosis, causing abnormal sperm morphology and reducing sperm count and motility.^32^ To the best of our knowledge, the current study is the first that investigated the predictors of successful sperm retrieval in men with NOA living in an Arabic oversea hypoxia city that predispose them to chronic environmental hypoxia exposure.

The present study is a retrospective one that included a small number of cases. In addition, it was conducted at one center, causing a limitation to our study. However, more prospective studies on a large population could be advised.

## 5 CONCLUSION

The successful extraction of spermatozoa in men with NOA using a surgical retrieval technique is relatively high, with good pregnancy and live birth rates. Patients' age and serum FSH level are independent predictors of successful sperm retrieval for men with NOA; young patients with high FSH levels could have little chance of sperm retrieval.

Testicular histopathology is not a significant predictor of successful sperm retrieval in men with NOA. Nevertheless, hypospermatogenesis has the best prognosis.

## AUTHOR CONTRIBUTIONS

**Abdulkareem Aljubran**: writing—original draft; writing—review and editing. **Omar Safar**: investigation; writing—original draft; writing—review and editing. **Adel Elatreisy**: formal analysis; writing—original draft; writing—review and editing. **Raed Alwadai**: investigation; writing—original draft. **Osama Shalkamy**: writing—review and editing. **Hassan Mohammed Assiri**: writing—review and editing. **Mamdoh Eskander**: supervision; writing—review and editing. **Adel Arezki**: writing—review and editing. **Ahmed Ibrahim**: writing—original draft; writing—review and editing.

## CONFLICTS OF INTEREST

The authors declare no conflicts of interest.

## TRANSPARENCY STATEMENT

I Omar Safar, affirms that this manuscript is an honest, accurate, and transparent account of the study being reported; that no important aspects of the study have been omitted; and that any discrepancies from the study as planned.

## ETHICS STATEMENT

Ethical Ref No.: UBCOM/H‐06‐BH‐087.

## Data Availability

The data that support the finding of this study are available from the corresponding author upon request.
